# Using evidence in mental health policy agenda-setting in low- and middle-income countries: a conceptual meta-framework from a scoping umbrella review

**DOI:** 10.1093/heapol/czad038

**Published:** 2023-06-17

**Authors:** Chloe Brooks, Tolib Mirzoev, Diptarup Chowdhury, Sonia Pereira Deuri, Anna Madill

**Affiliations:** School of Psychology, University of Leeds, Lifton Terrace, Leeds LS2 9JT, UK; Department of Global Health and Development, London School of Hygiene and Tropical Medicine, London, UK; Department of Clinical Psychology, Lokopriya Gopinath Bordoloi Regional Institute of Mental Health, Tezpur, Assam 784001, India; Department of Psychiatric Social Work, Lokopriya Gopinath Bordoloi Regional Institute of Mental Health, Tezpur, Assam 784001, India; School of Psychology, University of Leeds, Lifton Terrace, Leeds LS2 9JT, UK

**Keywords:** Review, mental health, policy, framework, evidence-based policy, agenda-setting

## Abstract

The purpose of this article is to close the gap in frameworks for the use of evidence in the mental health policy agenda-setting in low- and middle-income countries (LMICs). Agenda-setting is important because mental health remains a culturally sensitive and neglected issue in LMICs. Moreover, effective evidence-informed agenda-setting can help achieve, and sustain, the status of mental health as a policy priority in these low-resource contexts. A scoping ‘review of reviews’ of evidence-to-policy frameworks was conducted, which followed preferred reporting items for systematic reviews and meta-analyses (PRISMA) guidelines. Nineteen reviews met the inclusion criteria. A meta-framework was developed from analysis and narrative synthesis of these 19 reviews, which integrates the key elements identified across studies. It comprises the concepts of evidence, actors, process, context and approach, which are linked via the cross-cutting dimensions of beliefs, values and interests; capacity; power and politics; and trust and relationships. Five accompanying questions act as a guide for applying the meta-framework with relevance to mental health agenda-setting in LMICs. This is a novel and integrative meta-framework for mental health policy agenda-setting in LMICs and, as such, an important contribution to this under-researched area. Two major recommendations are identified from the development of the framework to enhance its implementation. First, given the paucity of formal evidence on mental health in LMICs, informal evidence based on stakeholder experience could be better utilized in these contexts. Second, the use of evidence in mental health agenda-setting in LMICs would be enhanced by involving a broader range of stakeholders in generating, communicating and promoting relevant information.

Key messages

- There are multiple frameworks for understanding, strengthening and assessing the role of evidence in health policy-making, including to some extent agenda-setting. However, there is paucity of frameworks specifically for mental health agenda-setting in low- and middle-income countries (LMICs).
- Current frameworks predominantly, and often implicitly, emphasize (i) research evidence and omit informal evidence such as expert advice and community narratives and (ii) professional actors such as researchers, policymakers and practitioners, rather than communities and civil society.
- Effective use of evidence for mental health agenda-setting in LMICs should be cognisant of (i) less abundance of formal research evidence; (ii) the stigmatization surrounding mental health, which may exclude those affected from participation in policy-making and agenda-setting processes, (iii) different kinds of policy actors that have unequal power in these processes.
- A meta-framework is presented with specific application to mental health agenda-setting in LMICs, along with practical recommendations for its implementation. It comprises the concepts of evidence, actors, process, context and approach, which are linked via the cross-cutting dimensions of beliefs, values and interests; capacity; politics and power; and trust and relationships.

## Introduction

Evidence-informed policy-making occurs when governments base their policies and plans on the best available information (Green and Bennett, 2007; Brownson *et al.*, 2009). The use of evidence to inform policy-making offers the best chance that actions address the needs of the population and with efficiency of public expenditure (Allen, 2017), particularly important in low-resource settings.

Recognized as a global development priority (Patel *et al.*, 2018), mental health policy provision is receiving greater attention, particularly in the context of low- and middle-income countries (LMICs). This is demonstrated by the increase in the prominence of mental health within the Sustainable Development Goals compared to the preceding Millennium Development Goals (Mills, 2018). Targets (3.4, 3.5 and 3.8) for SDG3 ‘Good Health and Well-Being’ (United Nations, 2015) all relate to mental health.

The Stages Heuristic Model is the prevailing conceptualization of the policy-making cycle (Walt *et al.*, 2008). Four stages are posited: agenda-setting, policy formulation, implementation and evaluation. Agenda-setting is the focus of the present study given that mental health remains a sensitive and stigmatized issue (Thornicroft *et al.*, 2022), globally and in LMICs (Javed *et al.*, 2021), leading to relatively limited political attention and under-prioritization of mental health on the policy agenda. This, in turn, leads to the absence, or ineffective implementation, of mental health policies in many LMICs (Omar *et al.*, 2010; Bird *et al.*, 2011), The coronavirus disease (COVID)-19 pandemic has given rise to a ‘global crisis for mental health’ (World Health Organization, 2022, para 3) with long-term and profound effects most acute in low-resource settings (Kola *et al.*, 2021). As well as shining a light on mental health as a policy issue in LMICs (Torales *et al.*, 2020), the pandemic has shifted societal priorities, processes and roles (Kola *et al.*, 2021). Consequently, there is a unique opportunity to scale-up and reshape the agenda for mental health and understanding of the evidence by which it is informed (Goldman *et al.*, 2020). Effective evidence-informed agenda-setting can be particularly instrumental in bringing and maintaining mental health as a priority policy issue. However, the pervasive stigma surrounding mental health globally has also been proposed to provide a challenge to the use of evidence for mental health policy-making (Botticelli, 2019).

Many LMICs lack a stand-alone mental health policy, and one-quarter (25%) of World Health Organization (WHO) member states do not have a stand-alone mental health policy or plan (World Health Organization, 2021b). Existing policies are not always evidence informed (Omar *et al.*, 2010; Williamson *et al.*, 2015). For example, in Commonwealth countries with a mental health policy, only 8% refer explicitly to within-country data and to research that informed policy development (Bhugra *et al.*, 2018). Furthermore, the research that exists is often not being used to inform policy in LMICs (Wei, 2008; Williamson *et al.*, 2015). Indeed, it has been argued for health policy more broadly that the lack of translation of evidence into policy-making is as important a focus as bridging than the evidence gap (Martin *et al.*, 2019; World Health Organization, 2021a).

Substantial headway is being made in conceptualizing the intricate relationship between evidence and policy (Smith and Joyce, 2012). Theory is particularly useful for health systems and policy research (de Leeuw *et al.*, 2014), due to the complexity involved (Gilson, 2012). Specifically, frameworks can provide a structure within which to organize and describe the relationship between variables (Nilsen, 2015). Moreover, frameworks provide a scaffold on which theory can be synthesized and summarized to aid application (Kivunja, 2018) and thus shape and structure inquiry (Walt *et al.*, 2008). For example, the taxonomy of models of evidence use comprising incrementalist, rational and networks (Hanney *et al.*, 2003) can help explain the nature of engagements of different policy actors in health policy processes and possibly inform strategies for enhancing evidence use in health policy-making.

An initial scoping search revealed numerous frameworks for understanding, strengthening and assessing the role of evidence in health policy-making more generally. Yet, only one framework focused specifically on mental health agenda-setting in LMICs: the EVIdence To Agenda setting (EVITA) framework (Votruba *et al.*, 2020; 2021). However, EVITA narrowly focuses only on research evidence. Evidence comes in a multitude of forms including both formal evidence produced by scientific research, such as academic studies and national surveys, as well as informal evidence based upon personal experience, such as expert opinion and community narratives (Mirzoev *et al.*, 2013; 2017). Focusing on published academic literature as the only evidence may exclude a large body of tacit knowledge and voices, from policy-making (Abimbola, 2021). Hence, it could be argued that a focus on formal mental health research evidence may prevent effective agenda-setting for mental health and reduce mental health inequities. Furthermore, empirical work on the role of evidence in mental health policy-making has also focused on research evidence (Williamson *et al.*, 2015).

In the current article, we reframe the focus to encompass a broader range of evidence, informal as well as formal. This is poignant for mental health policy-making due to the widely documented lack of formal mental health research evidence on many key topics relevant to government planning, including health provision and the creation of health policy (Omar *et al.*, 2010; Mackenzie, 2014; World Health Organization, 2018; Iemmi, 2022). This evidence gap appears to be most acute in LMICs; the percent of mental health research output against total research output (World Health Organization, 2021b) is lower in LMICs than in high income countries. Although mental health research output is increasing, it is in fact decreasing in comparison to general health research output (World Health Organization, 2021b).

In focusing on evidence, we also acknowledge that it is one of many influences on policy decisions, which are taken by policy actors who typically bring their agendas and interests and are engaged in complex power interplay (Walt and Gilson, 1994; Capano and Malandrino, 2022). Exploring the wide range of influences on mental health policy decisions is outside the scope of this study, but in addition to examining the role of evidence per se, we also explore the, perhaps equally important, inter-related contextual elements that affect the (non)use of evidence.

The context is especially important in relation to mental health (Montenegro and Ortega, 2020) given heterogeneity of local understandings and implications (Krendl and Pescosolido, 2020), including stigma (Gopalkrishnan, 2018). Seeking universal applicability may potentially reduce and distort complex realities (Abimbola, 2021). The EVITA framework has to date been applied to the South African and the global LMIC context (Votruba *et al.*, 2021). Votruba et al. (2018) argued that frameworks from other health/policy areas can offer lessons for strengthening the role of evidence in mental health agenda-setting in LMICs and the value of synthesizing learning across settings in relation to evidence-informed policy-making has been demonstrated (Langlois *et al.*, 2016).

Hence, it is appropriate to survey frameworks from the wider health policy literature for insights (Buse, 2008). However, the insights from general health frameworks and frameworks from other areas are limited as they do not capture the unique context of mental health policy and evidence use.

The aim of this article is to report results of a ‘review of reviews’ of evidence to health policy frameworks to glean insights into mental health agenda-setting in LMICs. Our review sought to answer the following research question: What can be learnt from health evidence-to-policy frameworks for the use of evidence in mental health agenda-setting in LMICs? The first objective of this review was to review the applicability of current theorizations and frameworks for evidence-informed policy-making to mental health agenda-setting in LMICs. A second objective was, as a result, to propose a meta-framework for the role of evidence in agenda-setting for mental health policy-making in LMICs. We hope that this article will be of interest and relevance to policymakers, practitioners and researchers who are interested in advancing the understanding of and improving evidence-informed mental health agenda-setting and improving evidence-informed policy-making more generally.

## Methods

### Review of reviews

Given the existence of multiple reviews of health evidence-to-policy frameworks (e.g. Graham *et al.*, 2007; Ward *et al.*, 2009), instead of reviewing primary sources, we conducted a scoping ‘review of reviews’ (Smith *et al.*, 2011), also referred to as ‘overview of reviews’ (Hunt *et al.*, 2018) or ‘umbrella review’ (Aromataris *et al.*, 2015), following the PRISMA guidelines. Reviews of reviews are a relatively recently established and distinct form of evidence synthesis, which aim to integrate the findings of different reviews on the same topic (Oliver *et al.*, 2014). Comparing and contrasting the findings of individual reviews enable the assessment of the consistency of research findings, identification of ambiguities and discovery of insights adding value beyond restating previous findings (Hasanpoor *et al.*, 2019). Reviews of reviews are particularly beneficial where there are multiple reviews of the same topic that differ in quality, scope and exact focus. Our approach allows us to identify relevant theories, assess their importance and offer a synthesis with respect to evidence to health policy frameworks to glean insights into mental health agenda-setting in LMICs (Campbell *et al.*, 2014).

### Search strategy

#### Database selection

Four health-related academic databases were searched in November 2018 with alerts for later relevant publications until October 2022 when the review was largely completed: Medline, Global Health, Health Management Information Consortium (HMIC) and PsychINFO, followed by citation search for further publications. The HMIC database includes grey literature (Paez, 2017), which increased the comprehensiveness of our review.

#### Search terms

The BeHEMoTh framework (Booth and Carroll, 2015b) was used to define the key components of the research question (Table 1). A concept map was subsequently developed setting out the search terms for the database search, consisting of review, frameworks, evidence, policy-making, and the pathway of evidence-to-policy. It was particularly challenging to devise adequate search terms for the latter given the large number of potential synonyms (McKibbon *et al.*, 2010) and low specificity due to the inclusion of these terms in policy-relevant papers. The search strategy (Supplementary Information 1) was only modified for each database where required for technical reasons such as differences in subject headings.

**Table 1.** BeHEMoTh framework for specification of theory-related review questions and its application to the present review

	BeHEMoTh framework	Applied to present review
Be	Behaviour of interest: way population or patient interacts with health context	Evidence-to-policy
H	Health context: service, policy, programme or intervention	Health policy (including agenda-setting, formulation, implementation and evaluation)
E	Exclusions: nontheoretical/technical models, depending on volume	Nontheoretical models
MoTh	Models/theories: model, theory, concept or framework strategy	Underlying theories will be analysed, but reviews of frameworks are the focus. Search will include reviews of models due to the inconsistent terminology.

#### Inclusion criteria

Our inclusion criteria were the existence of theoretical/conceptual frameworks in the review; focus on the role of evidence and the process of health policy-making; published in English and published in or after 2004. Although we are primarily interested in the agenda-setting stage of the policy-making cycle and/or LMIC contexts, our scoping search suggested that such a narrow focus in the first instance would yield insufficient results to elicit meaningful findings. We followed the definition of frameworks as structures that describe the relationship between variables (Nilsen, 2015). Results were limited to reviews published in or after 2004 because this was the year of the landmark WHO Mexico Ministerial Health Summit (The Lancet, 2004), which increased attention to evidence-informed policy-making (Bennett *et al.*, 2018).

### Screening and quality assessment

Results were first screened by titles and abstracts, then full texts. Results were single-screened, and the screener brought unresolved questions regarding individual papers to the team for discussion and joint decision-making. Screening mainly filtered out results that were not reviews of theoretical/conceptual frameworks due to the difficulties in designing a search with high specificity. There was minimal ambiguity and any unclear decisions were discussed among all researchers.

Limited tools exist specifically for assessing the quality of reviews of frameworks or theory. Therefore, an adapted version of GRADE-CERQual was used (Supplementary Information 2). GRADE-CERQual provides an assessment of confidence in the evidence from systematic reviews of qualitative research or syntheses of qualitative evidence (Lewin *et al.*, 2018) and is used widely (Pollock *et al.*, 2020). GRADE-CERQual is suitable for adaptation because it can incorporate other tools: we incorporated Critical Appraisals Skills Programme (CASP) (CASP, 2018) and A MeaSurement Tool to Assess systematic Reviews (AMSTAR 2) (Shea *et al.*, 2017).

The developers of GRADE-CERQual recommend assessing the primary studies included in each review (Munthe-Kaas *et al.*, 2018). We were not able to do this for the following reasons: limited tools and guidance are available for assessing the quality of theoretical papers (Votruba *et al.*, 2018); the primary studies in the selected review articles do not allow meaningful application of more established tools (Contandriopoulos *et al.*, 2010); and authors, on the whole, did not attempt to assess the quality of their primary studies. The present study is interested in the quality of the reviews themselves, in how well they synthesize findings, and less on quality of the primary studies. Hence, if a review provides insufficient details on primary studies to enable GRADE-CERQual assessment, or presents an appropriate quality appraisal, these are notable findings. As a consequence, rather than assessing the level of confidence that can be placed in the body of data directly, we have assessed how reviews have evaluated the quality of primary studies included in their own review and considered the confidence that can be placed in the conclusions that authors have drawn from their findings.

All included reviews were scored independently by the first author and an independent assessor. Inter-rater reliability was high: 15 of the 19 reviews were given the same rating (80%). Three reviews were discrepant in only a single rating, and one review was discrepant in two ratings. All disagreements were resolved at a consensus meeting. About half the reviews (9 out of 19, 47%) were awarded a high level of confidence in their findings: the highest-level possible (Supplementary Information 3). Only one review (5%) was awarded the lowest level of confidence (very low). Following the GRADE-CERQual approach, no review was excluded. However, the score was considered in interpreting results of our analysis. Few reviews sufficiently assessed and/or documented the quality of the frameworks or the primary studies. Additionally, few reported the source of funding of the frameworks, or studies. Frameworks produced outside of academia, for example, by nongovernmental organizations, may be more likely to use a broader definition of ‘evidence’ beyond research.

### Analysis and synthesis of results

For the review papers included in the sample, the full article was analysed with the narrative synthesis of the reviews and any frameworks produced from the reviews as data. Data were extracted by a single author into the data extraction table. For the analysis of our data and synthesis of results, we were guided by the ‘best fit’ framework synthesis approach (Carroll *et al.*, 2013): an established method for the systematic review of qualitative evidence (Booth and Carroll, 2015a; Brunton *et al.*, 2020). An existing framework from the literature, devised for a closely related purpose, is used as a starting point to aid the initial analysis of the data. Through the analysis, the initial framework is developed; new concepts that cannot be incorporated can also be generated, thereby creating a new ‘meta-framework’ based on the a priori concepts and expanded with any new concepts (Carroll *et al.*, 2013).

An obvious candidate for the initial a priori framework was the policy triangle (Walt and Gilson, 1994), a general framework ubiquitous in health policy analysis. Not overly prescriptive, the policy triangle is often used alongside other frameworks (O’Brien *et al.*, 2020). Moreover, the policy triangle was designed primarily for health policy reform in LMIC settings (O’Brien *et al.*, 2020).

**Figure 1.**
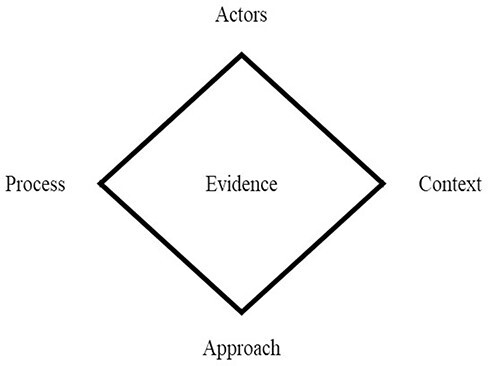
The initial framework used in the analysis

The policy triangle consists of four concepts: actors, context, content and process. At the outset, we replaced ‘content’ with ‘evidence’ due to our specific interest in how evidence informs policy. During the analysis, it was apparent that a fifth concept, ‘approach,’ was needed to capture this aspect of our findings and enable actionable recommendations. The resultant concepts that form the initial framework (Figure 1) are defined in the Results section.

Thematic analysis was applied to identify patterns in the data (Braun and Clarke, 2006) to enable the frameworks to be compared and contrasted. After familiarization, data were coded inductively under the concepts of the a priori framework. The codes were then grouped together to form higher-level descriptive factors, both common and unique, which were iteratively developed. Higher-level interpretation of these descriptive factors enabled the concepts of the framework to be linked together.

Multiple links between the different concepts were explored, with the data being continually revisited to ensure that the data supported the links and the significance ascribed to them. These links were used to extend the initial framework and link the concepts together, with a meta-framework being developed from the multitude of frameworks. At regular stages, the authors critically discussed the analysis to ensure that the framework represented the data and was as useful to the intended audience. This meta-framework was then considered alongside existing knowledge about the mental health agenda-setting in LMICs to explore how the framework might be usefully tailored to this specific context.

An approach that combines inductive and deductive analysis and starting with predetermined concepts was appropriate because health evidence-to-policy has been previously studied by various scholars (Oliver *et al.*, 2014). Hence, it was reasonable to anticipate that concepts suggested by classic previous literature will be relevant. This helped us connect our findings to the extant research while adding nuance through iterative refinement and development of these concepts via inductive analysis of the data. Our ‘review of reviews’ therefore unites common and unique elements of existing frameworks into a meta-framework.

## Results

The next sections will explore the included reviews, including the key factors identified for the use of evidence, culminating in the development of a meta-framework for the role in evidence in agenda-setting for mental health policy-making in LMICs.

### Overview of included reviews

**Figure 2.**
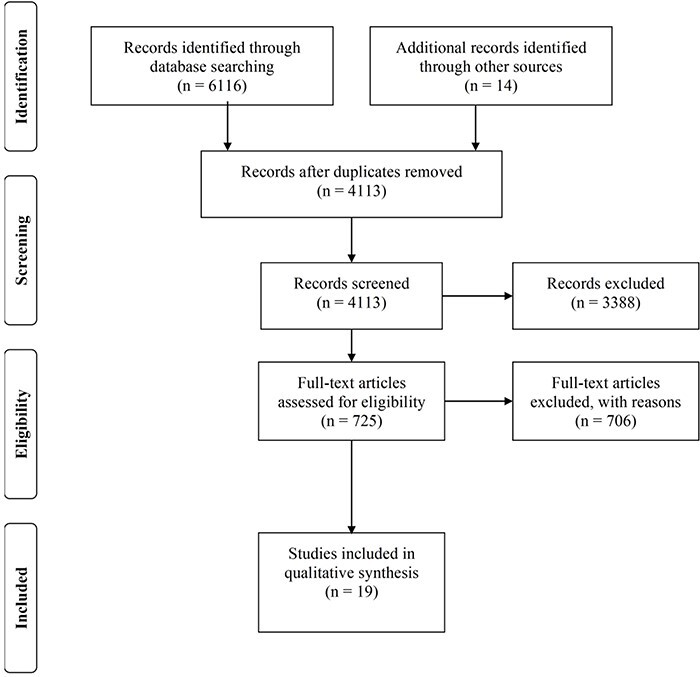
PRISMA flow diagram

The PRISMA flow diagram (Figure 2) shows that the initial database search yielded 6116 articles. A further 32 articles were included from the citation search, and 1060 duplicates were removed. After title and abstract screening, 725 articles were retained and the full texts were assessed for eligibility. Nineteen met the inclusion criteria (Supplementary Information 3). No eligible reviews were identified via alerts after the initial search in November 2018.

The two reviews focused on ‘assessing’ the use of evidence in health policy-making (Cruz Rivera *et al.*, 2017; Newson *et al.*, 2018) identified were not analysed further due to the limited relevance for mental health in LMICs.

An adapted version of GRADE-CERQual that was developed to provide an assessment of confidence in the evidence from systematic reviews of qualitative research or syntheses of qualitative evidence (Lewin *et al.*, 2018) was used (see the Screening and quality assessment section). The reviews included both systematic (*N* = 6; 32%) and nonsystematic narrative reviews (*N* = 13; 68%). Greenhalgh et al. (2018) argued that narrative reviews should not be considered lower in the evidence hierarchy that systematic reviews and, although our quality appraisal tended to assign them a lower level of confidence, some narrative reviews were rated ‘high’.

The only review not authored from the Global North was by Almeida and Báscolo (2006) situated in Brazil and Argentina. The dominance of authorship from the Global North was also reflected for the individual frameworks (Votruba *et al.*, 2018).

Four reviews originated from a particular area of health: health surveillance (Green *et al.*, 2009), nursing (Mitchell *et al.*, 2010), emergency medicine (Graham *et al.*, 2007) and mental health (Votruba *et al.*, 2018). Nonetheless, all studies reviewed general health evidence-to-policy frameworks. Votruba et al. (2018) limited their review to frameworks that had been applied to mental health policy-making in an LMIC setting.

Some reviews built upon previous reviews. For example, Damschroder et al. (2009) used the findings of Greenhalgh et al. (2004). In turn, Moullin et al. (2015) built on Damschroder et al. (2009) to produce their framework. Ten of the 17 reviews listed included frameworks in an easily accessible tabular format. Analysis of this subset indicates a reasonably high percentage of unique frameworks in each review (18%—59%, Table 2). Variation in the foci and inclusion criteria of the reviews does not fully explain why frameworks are included in some, but not other, reviews. Moreover, some reviews treated different versions of the same framework as distinct, while others considered the different versions combined.

**Table 2.** Proportion of unique frameworks within each review

	Review	frameworks not included by other reviews within this subset^a^ (%)
(1)	Ward et al. (2009)	9/18 (50)
(2)	Damschroder et al. (2009)	5/19 (26.3)
(3)	Mitton et al. (2007)	2/5 (40)
(4)	Votruba et al. (2018)	3/4 (75)
(5)	Milat and Li (2017)	24/41 (58.5)
(6)	Moullin et al. (2015)	23/49 (46.9)
(7)	Tabak et al. (2012)	11/61 (18.0)
(8)	Mitchell et al. (2010)	11/47 (23.4)
(9)	Wilson et al. (2010)	13/33 (39.3)
(10)	Nilsen (2015)	13/35 (37.1)

a The subset of reviews used for this piece of analysis were reviews that focused on frameworks to explain or strengthen (not assess) the use of evidence in health policy-making and listed studies in an easily accessible tabular format.

**Figure 3.**
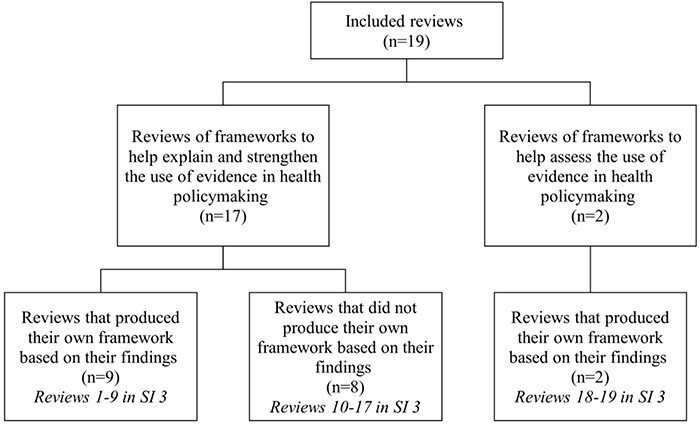
Summary of included reviews

Each review did one or more of the following: described, categorized, compared and contrasted (including from different fields), and critiqued existing frameworks of (at least some part of) the evidence-to-policy pathway. The level of details provided on included frameworks varied greatly as did the level of analysis. Some reviews presented a list of available frameworks, some provided a categorization and some identified common factors. Seventeen focused on ‘explaining’ and ‘strengthening’ the use of evidence in health policy-making (Figure 3). Of these, eight provided a synthesis to summarize the development of the current evidence base and to aid the selection of relevant frameworks and nine produced a new framework intended to guide action, research and discussion. These 17 reviews were analysed to identify which of our priori key concepts were included in the synthesis or framework produced (the full analysis is provided in the Supplementary Information). ‘Actors’ were a major concept in the lowest number of reviews (47%), with ‘approach’ included by the greatest number of reviews (77%). This suggests that developing recommendations for strengthening the role of evidence is a key focus.

### Underlying theories

Theories explain the relationship between variables, whereas frameworks are structures to describe the relationship between variables (Nilsen, 2015). Thus, it is useful to understand the theories that underpin frameworks, to help assess the applicability of health evidence-to-policy frameworks to mental health policy agenda-setting in LMICs.

The theories underlying individual frameworks included in each review were rarely presented. Mitton et al. (2007) was an exception. Most reviews noted that some frameworks were based on existing theories, others on empirical studies and some on the authors’ personal experience. The nine reviews that produced a new framework were analysed to understand what theories contributed to their development, and this is presented in Table 3. Relevant information was often alluded to indirectly but was sometimes dealt with explicitly, for example, in the discussion.

**Table 3.** Key theories apparent in the frameworks produced by the reviews

		Theory of diffusion of innovations (Rogers, 2010)	Two communities theory of research utilization (Caplan, 1979)	Theory of opinion leadership (Katz and Lazarsfeld, 1955)	Social network theory (Barnes, 1954)	Complex system theory (Thompson *et al.*, 2016)	Punctuated equilibrium Theory (Baumgartner and Jones, 1991)
Reviews that produced a framework (*N* = 9)							
		Encompasses categories of adopters (innovators, early adopters, early majority, late majority and laggards), the stages of adoption of an innovation (awareness, decision to adopt, initial use and continued use) and factors that influence adoption (relative advantage, compatibility, complexity, trialability and observability)	Argues that researchers and policymakers reside in distinct spheres, and there is a gap that needs to be bridged.	States that messages reach users via ‘opinion leaders’, other users with influence, which interpret the information and pass on this information as well as their interpretation.	Actors are represented by nodes, and the relationships between them are represented as ties. The relationships between actors are viewed to be more important than the characteristics of individual actors.	For complex systems, the system is greater than the sum of its individual components and therefore must be studied as a whole. As a small action can affect the whole of the system due to feedback mechanisms, it is difficult to make predictions and thus unintended consequences can occur.	Rather than undergoing gradual change, policies experience long periods of stability that are interspersed with shorter periods of dramatic change.
(1)	Contandriopoulos *et al.* (2010)	X	✓	X	✓	✓	X
(2)	Damschroder *et al.* (2009)	✓ Categories of adopters Factors that influence adoption	X	✓	✓	✓	X
(3)	Gold (2009)	X	✓	✓	✓	X	✓
(4)	Graham *et al.* (2007; 2006)	X	X	X	X	X	X
(5)	Green *et al.* (2009)	✓ Factors that influence adoption	X	✓	X	X	X
(6)	Greenhalgh *et al.* (2004; 2007)	✓ Categories of adopters Stages of adoption Factors that influence adopters	X	✓	✓	✓	X
(7)	Moullin *et al.* (2015)	✓ Stages of adoption Factors that influence adopters	X	X	✓	✓	X
(8)	Votruba *et al.* (2018)	X	✓	✓	✓	X	✓
(9)	Ward *et al.* (2009)	✓ Factors that influence adoption	✓	X	X	✓	C
Total (% of reviews)		5 (55.6%)	4 (44.4%)	5 (55.6%)	6 (66.7%)	5 (55.6%)	2 (22.2%)

Six key theories, apparent in the frameworks produced by the reviews, were identified: Theory of Diffusion of Innovations (*N* = 5, 56%) (Rogers, 2010); Two Communities Theory of Research Utilization (*N* = 4, 44%) (Caplan, 1979); Theory of Opinion Leadership (*N* = 5, 56%) (Katz and Lazarsfeld, 1955); Social Network Theory (*N* = 6, 67%) (Barnes, 1954); Complex System Theory, or Complexity Theory (*N* = 5, 56%) (Thompson *et al.*, 2016); and Punctuated Equilibrium Theory (*N* = 2, 22%) (Baumgartner and Jones, 1991). All six theories originated outside of health policy. Punctuated Equilibrium Theory and the Two Communities Theory both have their origins in the field of political science and public policy. The Theory of Opinion Leadership was developed in media and communication sciences. Social Network Theory came from social and behaviour sciences. Complex System Theory is transdisciplinary. None of the nine new frameworks appeared to be influenced by all six theories. Notably, in many frameworks, the Two Communities Theory was extended to include three communities: researchers, policy-makers and intermediaries.

### Relevance to mental health agenda-setting in LMICs

There was broad consensus among the reviews that few frameworks have been applied and tested for any context (Ward *et al.*, 2009; Votruba *et al.*, 2018). However, the extent to which the application of frameworks was explored by reviews varied with some, for example, offering citation frequency (Tabak *et al.*, 2012). Application of frameworks to mental health policy-making in LMICs was even more limited (Votruba *et al.*, 2018). In addition to the four frameworks identified by (Shiffman and Smith, 2007), the framework by Votruba et al. (2018) was noted to have been applied to global mental health and to be relevant to evidence, although focused on the determination of issue salience (Tomlinson and Lund, 2012).

Interestingly, power and political context feature strongly in the frameworks identified by Votruba *et al.* (2018) to have been applied to mental health in LMICs, including the Rapid framework (Court and Young, 2006), and the Knowledge Policy and Power framework (Jones *et al.*, 2013). This indicates the perceived importance of these factors to mental health by authors selecting appropriate frameworks. Key findings with regard to the application of the frameworks include the need for early stakeholder engagement, to understand the beliefs and values of actors and to integrate monitoring and evaluation to assess the use of evidence in policy-making. Reflection on the utility of the frameworks was limited, although the authors were always positive. Tomlinson and Lund (2012) propose that a debate among researchers is needed to agree upon key policy priorities and solutions for mental health in order to advocate more coherently and convince policymakers to take action based upon the evidence presented. The only suggested refinement was greater consideration of the heterogeneity of mental health (Mackenzie, 2014) due to the challenges this presents to constructing a single clear policy ask.

The findings from our thematic analysis will now be presented under the five key concepts (evidence, actors, process, context and approach). The specific relevance of these findings for mental health policy agenda-setting in LMICs will also be highlighted. Table 4 shows the extent to which the reviews focused upon each of the concepts.

**Table 4.** Concepts of the new frameworks produced by the reviews or considered by the reviews where a new framework was not produced

		Evidence	Actors	Process	Context	Approach
Reviews that produced a new framework (*N* = 9)						
(1)	Contandriopoulos *et al.* (2010)	✓	✓	X	✓	✓
(2)	Damschroder *et al.* (2009)	✓	✓	✓	✓	✓
(3)	Gold (2009)	✓	✓	✓	✓	✓
(4)	Graham *et al.* (2007; 2006)	✓	X	✓	X	X
(5)	Green *et al.* (2009)	✓		✓	✓	X
(6)	Greenhalgh *et al.* (2004; 2007)	X	✓	✓	✓	✓
(7)	Moullin *et al.* (2015)	X	X	✓	✓	✓
(8)	Votruba *et al.* (2018)	✓	✓	✓	✓	✓
(9)	Ward *et al.* (2009)	✓	X	✓	✓	✓
Reviews that did not produce a new framework (*N* = 8)						
(10)	Almeida and Báscolo (2006)	✓	✓	✓	✓	✓
(11)	Milat and Li (2017)	X	X	X	✓	X
(12)	Mitchell *et al.* (2010)	X	x	✓		✓
(13)	Mitton *et al.* (2007)	X	X	X	X	
(14)	Nilsen (2015)	✓	✓	✓	✓	✓
(15)	Oborn *et al.* (2013)	✓	✓	✓	✓	✓
(16)	Tabak *et al.* (2012)	X	X	X	✓	✓
(17)	Wilson *et al.* (2010)	X	X		X	✓
Total		10 (58.8%)	8 (47.1%)	12 (70.6%)	13 (76.5%)	13 (76.5%)

### Evidence for mental health in agenda-setting

Findings emerged from our analysis related to evidence in four key areas: nature, perception, supply and demand, and use.

#### Nature of evidence

‘Evidence’ was often used interchangeably with related terms such as ‘knowledge’ and not explicitly defined. Different types of evidence were identified including ‘tacit’, ‘implicit’, and ‘explicit’ (Oborn *et al.*, 2013). Evidence from ‘formal’ research was generally prioritized over ‘informal’ sources, such as expert opinion. Because of the mental health evidence gap, especially in LMICs (Omar *et al.*, 2010; Mackenzie, 2014; World Health Organization, 2018), ‘informal’ sources may be particularly important. One review discussed how research evidence originating from different ‘disciplines’ is perceived differently, with the social sciences sometimes viewed as providing ‘shallow’ insights (Contandriopoulos *et al.*, 2010). Finally, some reviews highlighted a need to understand how research evidence is considered and integrated alongside other sources of information (Almeida and Báscolo, 2006; Contandriopoulos *et al.*, 2010).

Important characteristics identified for evidence, although not specifically for agenda-setting, surrounded the context of its intended use and include ‘relevance’, ‘applicability’ and ‘salience’ (Gold, 2009). Accordingly, the capacity of stakeholders to appraise the ‘quality’ and ‘value’ of evidence featured in the reviews (Mitton *et al.*, 2007; Damschroder *et al.*, 2009; Green *et al.*, 2009). This is particularly important in mental health agenda-setting to avoid stigma-related prejudice introducing bias, and knowledge synthesis is considered a useful mechanism to improve the robustness of evidence (Graham *et al.*, 2007).

#### Perception of evidence

As one review noted, evidence is encountered often in a social context and is open to debate and interpretation (Oborn *et al.*, 2013), influenced by the beliefs, values and biases of the audience. As argued elsewhere, destigmatizing of mental health therefore warrants greater focus (Botticelli, 2019). Reviews tended to focus on how policymakers and researchers may interpret the evidence differently. One review highlighted how discrepancies between researchers can undermine confidence in the evidence (Almeida and Báscolo, 2006).

For mental health agenda-setting in LMICs, the influence of stigma may mean that formal research evidence is actually viewed as more robust than informal evidence, based on personal experience, that comes directly from communities (Mackenzie, 2014). Communities are also recognized as important users and sources of mental health evidence (World Health Organization, 2005), and therefore, understanding the factors that shape the perception of a wide-ranging array of stakeholders is likely to be useful given the important influence of different beliefs, values and biases.

#### Supply and demand of evidence

Supply and demand was often framed as the, dynamic, mismatch between the availability of evidence and demands of policymakers (Milat and Li, 2017). An area of exploration for mental health agenda-setting in LMICs is the evidence needs of other stakeholders, such as communities and service users. Information overload was raised as a potential challenge (Mitton *et al.*, 2007), although this may be less relevant for mental health in LMICs given the evidence gap (World Health Organization, 2021b).

#### Use of evidence

Different uses of evidence were recognized including ‘conceptual’, ‘direct’, ‘tactical’, ‘political’, ‘imposed’ and ‘procedural’ (Almeida and Báscolo, 2006; Gold, 2009; Oborn *et al.*, 2013; Votruba *et al.*, 2018). Prior identification of how the evidence is intended to be used was reported to be likely to increase the effectiveness with which evidence is communicated by better defining the intended audience defined and selecting the most appropriate medium (Graham *et al.*, 2007; Green *et al.*, 2011). The quality and quantity of evidence influence its utility in policy-making, often evaluated in terms of its practical value for policymakers rather than for the full range of stakeholders (Gold, 2009; Milat and Li, 2017). Moreover, evidence needs to be adapted to context (Mitchell *et al.*, 2010; Milat and Li, 2017) and premature use of research may have unintended negative consequences and ethical costs Graham et al. (2007). Hence, the availability of suitable evidence is a necessary but not sufficient condition for its use in policy-making.

### Actors who use evidence in mental health agenda-setting

Actors are individuals and groups directly or indirectly involved in policy-making. Interestingly, actors were the concept least featured by the reviews (Table 4). Three key factors related to actors were identified from the analysis: categories, characteristics and relationships.

The three predominant categories of actor identified were ‘researchers’ (producers of evidence), ‘policymakers’ (users of evidence) and ‘intermediaries’ (knowledge brokers). Some reviews acknowledged that their classifications were a gross simplification (Gold, 2009; Contandriopoulos *et al.*, 2010) and that the categories were not necessarily mutually exclusive (Gold, 2009). Other reviews, however, noted the large cultural differences between researchers and policymakers (Oborn *et al.*, 2013). Terminology sometimes implied a hierarchy of actors according to knowledge and expertise (Mitchell *et al.*, 2010). Interestingly, one review suggested that frameworks were often researcher-focused (Wilson *et al.*, 2010).

Characteristics of actors received attention in many frameworks. ‘Knowledge’ and ‘capacity’ were discussed within the context of the ability and power to use evidence (Contandriopoulos *et al.*, 2010; Moullin *et al.*, 2015). Capacity of individuals and organizations, including human and financial resources (Greenhalgh *et al.*, 2004). often constrained the ability of actors to use evidence in policy processes, including advocacy and agenda-setting (Votruba *et al.*, 2018). Although the focus tended to be on actors as individuals, their position within organizations and the characteristics of those organizations were reflected upon to varying degrees (Damschroder *et al.*, 2009; Contandriopoulos *et al.*, 2010).

Softer characteristics, including the ‘belief’, ‘values’ and interests of individual and organizational stakeholders, were a frequent factor (Greenhalgh *et al.*, 2004; Almeida and Báscolo, 2006; Mitton *et al.*, 2007; Damschroder *et al.*, 2009; Gold, 2009; Contandriopoulos *et al.*, 2010; Wilson et al. 2010; Nilsen, 2015; Votruba *et al.*, 2018). Beliefs, values and interests shape how actors understand the world, what they value as important and their interests and hence directly shape how evidence is used. Interestingly, in the review focused on frameworks for mental health (Votruba *et al.*, 2018), of the four relevant to LMICs only one has a component on actors’ beliefs, values and interests and are included only implicitly in the other three. Stigma against people with lived experience of mental health conditions is likely to affect how evidence on mental health is viewed and therefore used policy in agenda-setting (Botticelli, 2019).

Much of the conceptualization of the influence of beliefs, values and interests has come from outside the field of health policy (Jones *et al.*, 2013). The power and position of actors, including the power dynamics between actors, were important factors shaping the use of evidence.

The fit between actors and the relationships between them was viewed as potentially more important than their individual characteristics, with ‘trust’ being key. ‘Unequal power’ relations between stakeholders (Oborn *et al.*, 2013) alongside the ‘culture gap’, most frequently referred to between researchers and policymakers, were often noted to be barriers to good relationships. On the other hand, ‘long-term relationship building, bi-directional interaction and establishing stable networks’—both formal and informal—were argued to be conducive for strengthening the use of evidence in policy-making (Mitchell *et al.*, 2010; Oborn *et al.*, 2013). While the range of networks in relation to mental health policy-making may be restricted in LMICs, those that exist tend to be stronger than for other health policy issues (Mackenzie, 2014). It has been proposed that the widespread stigmatization of mental health has resulted in greater networking among people with lived experience of mental health conditions (Mackenzie, 2014). On the other hand, poor ‘financial investment’ in mental health can be a barrier to network activities and existence.

### The context in which actors use evidence in mental health agenda-setting

We define context as the setting in which actors make and implement policies and can include historical, political, economic and socio-cultural factors. Context was widely stated to be important, increasingly so in recent frameworks (Nilsen, 2015), although Milat and Li (2017) conclude that ‘real-world’ context is still lacking. Few reviews defined context, and it appeared to be used as a catch-all. Some divided the concept into levels (Greenhalgh *et al.*, 2004; Moullin *et al.*, 2015). Others cautioned that the boundary is not clearly defined, and the interaction between different aspects of context is important (Damschroder *et al.*, 2009). The key factors related to context identified from the analysis of the reviews are now presented under three levels: micro (individual level), meso (organization evel) and macro (systems level).

Micro-context (individual-level) often lacked detail, possibly because they appear less tangible and more difficult to assess than other contextual factors with regard to policy-making (Damschroder *et al.*, 2009). Due to the potential for stigma-related bias, micro-context in relation to mental health seems an area for greater framework development.

Meso-level (organization-level) factors centred on two components: ‘capacity’ covering ‘resources’ and ‘support’ and ‘motivation’ encompassing ‘culture’ and ‘leadership’ (Graham *et al.*, 2007; Mitton *et al.*, 2007; Moullin *et al.*, 2015; Votruba *et al.*, 2018). Damschroder et al. (2009) reflected on the importance of ‘interplay’ between individuals and organizations and highlighted this as an area needing more work.

The predominant macro-level (systems-level) contextual factors included in the reviews were ‘political and economic’ (Almeida and Báscolo, 2006; Contandriopoulos *et al.*, 2010). Broader ‘social’ and ‘cultural’ contexts, including language and socio-demographics, were reflected on, but to a lesser extent (Tabak *et al.*, 2012; Votruba *et al.*, 2018). ‘Technological’ context, such as digital connectivity (Tabak *et al.*, 2012), may be important yet under researched, particularly in relation to LMICs. The influence donor countries exert through development aid was noted in the review focused on mental health in LMICs (Votruba *et al.*, 2018), suggesting an area that may be missing from general health evidence-to-policy frameworks that largely originate from donor rather than recipient countries. Furthermore, mental health is often a cross-sectoral policy issue (Mackenzie, 2014) and this may broaden the contexts relevant to include.

### The process of mental health agenda-setting in which evidence is used

Policy process is the way in which policies are made and enacted, often conceptualized by the stages heuristic model as the stages of agenda-setting, development or formulation, implementation and evaluation (Walt *et al.*, 2008). Frameworks rarely focused on agenda-setting (Votruba *et al.*, 2018). The exception was Kingdon’s and Stano (1984) multiple streams framework (Tabak *et al.*, 2012) where issues rise to the top of the policy agenda when the problem, policy and politics streams converge. Although not solely focused on the role of evidence, the role of evidence in each can be considered. None of the four frameworks used for mental health LMICs identified by Votruba et al. (2018) specifically targeted the agenda-setting stage, pertinent for mental health due to the early stages of many policies.

Nevertheless, the complexity of the policy process was, however, still frequently emphasized. One review noted that newer frameworks gave greater recognition to this complexity (Almeida and Báscolo, 2006), although Gold (2009) concluded that frameworks still require greater detail. The ‘lengthiness’ and ‘unpredictability’ of the policy process were reported to present a challenge to the use of evidence (Graham *et al.*, 2007) due to the sustained investment of time and effort required, with no guarantee of a positive outcome. Additionally, many factors are often outside the influence of researchers (Gold, 2009).

Policy processes were seldom the sole explicit focus, and policy and practice frameworks were often grouped together (Tabak *et al.*, 2012). This is an important distinction as mental health policy-making, and particularly agenda-setting, is influenced by public perception (Bernardi, 2021), which for mental health is shaped by stigma.

Multiple terms were used to describe the movement of evidence, which was given greater emphasis than the policy process. Terms such as ‘translation’ (e.g Graham *et al.*, 2007) suggest a ‘unidirectional’ movement from evidence-to-policy, whereas ‘exchange’ (e.g. Contandriopoulos *et al.*, 2010) implies a ‘multidirectional’ process. Older frameworks more frequently conceptualized the evidence-to-policy process as ‘uni-directional’, suggesting a ‘supplier’ and a ‘receiver’ of evidence (Oborn *et al.*, 2013). Uni-directional models may therefore reinforce power differentials between actors. A bi-directional process, on the other hand, suggests a more equal distribution and transfer of evidence and therefore of power. ‘Nonlinear’, ‘multi-directional’ models emphasize interaction between researchers and policymakers and, in this way, tend to be people-centred (Ward *et al.*, 2009).

Power and politics was a recurring and cross-cutting factor that emerged from the analysis. The process of policy-making, and evidence-to-policy, is inherently political and shaped by the power dynamics between actors (Gore and Parker, 2019). Power and politics are closely related concepts; politics can be thought of the exercise of power. For mental health policy-making, this is particularly pertinent because people with lived experience of mental health conditions are often marginalized. Approaches that make more diverse kinds of evidence more widely available can help to redress power inequalities.

### Approaches to strengthen the use of evidence in mental health agenda-setting

Approaches are the means used to strengthen the role of evidence in policy-making. The extent to which reviews focused on the means (‘strategies’, ‘efforts’ and ‘activities’) used to strengthen the role of evidence in policy-making varied. Approaches could be categorized according to ‘effort (passive or active), direction (push or pull)and linkage (linear or bi/multidirectional) (Almeida and Báscolo, 2006). The reviews suggested that there is unlikely to be a singular best approach (Greenhalgh *et al.*, 2004) due to context, with a combination likely to be best (Gold, 2009). However, there was broad consensus that uni-directional communication was less likely to be successful, possibly due to the importance of interaction and dialogue between stakeholders (Almeida and Báscolo, 2006; Ward *et al.*, 2009; Contandriopoulos *et al.*, 2010; Mitchell *et al.*, 2010; Wilson *et al.*, 2010). The limitations of what can be influenced by researchers was also noted (Gold, 2009).

Tailoring approaches, including communication, to the intended audience was deemed critical (Greenhalgh *et al.*, 2004; Almeida and Báscolo, 2006; Mitton *et al.*, 2007; Wilson *et al.*, 2010). In LMICs, insufficient skill for communicating research has been documented, especially to nonspecialist audiences (Murunga *et al.*, 2020). This may be compounded in relation to mental health research because of cultural differences in the understanding of distress and disorder (Mackenzie, 2014).

Also important is the person delivering the message (Mitton *et al.*, 2007), and therefore, the relationships and trust between and within stakeholder groups (Mitton *et al.*, 2007; Contandriopoulos *et al.*, 2010; Wilson *et al.*, 2010) to create receptivity to the evidence and genuine relationships facilitate evidence generation, sharing, discussion and use. Reflecting the prominence of insights related to the behavioural sciences, their application to the policy-making process itself has received attention (Hallsworth *et al.*, 2018), recognizing that policy actors are prone to bias due to their human nature. However, incorporating these insights into approaches requires consideration of the context (Hallsworth *et al.*, 2018). Given the sensitive nature of mental health, trust between stakeholders is likely to be particularly important, especially when engaging marginalized communities who might be wary of researchers, medical professionals and policymakers.

### Meta-framework for the role of evidence in agenda-setting for mental health policy-making in LMICs

As described in the Introduction section, an objective of our review was to propose a meta-framework for the role of evidence in agenda-setting for mental health policy-making in LMICs. The main purpose of this framework is to draw upon the existing knowledge and advance understanding of evidence-informed mental health policy agenda-setting, through highlighting two somewhat neglected aspects: the importance of the context and a distinction between processes and approaches of evidence use.

**Figure 4.**
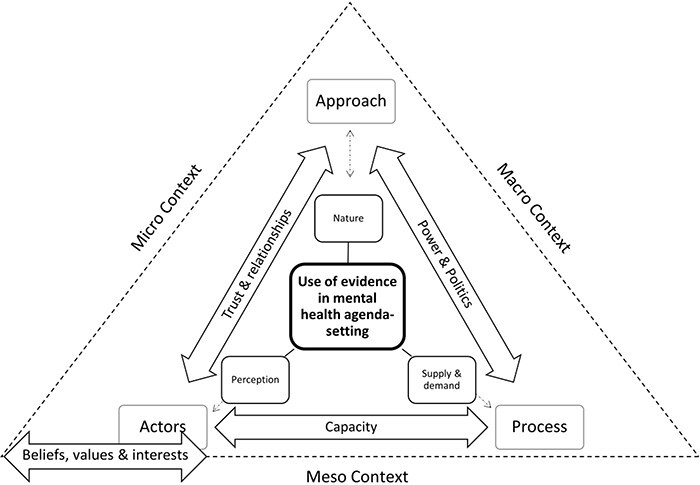
Meta-framework for the role of evidence in agenda-setting for mental health policy-making in LMICs

The use of evidence is multifactorial, and the availability of evidence is not sufficient to ensure its use in agenda-setting. Our framework (Figure 4) therefore differentiates five key inter-related concepts: evidence, actors, process, context and approach, which altogether determine the role of evidence in mental health agenda-setting, with the latter concept being key for ‘strengthening’ and not just understanding the use of evidence.

Given the focus of this study on the role of evidence, ‘use of evidence for mental health agenda-setting’ is naturally at the centre of our framework. The use of evidence is further distilled in our framework: the (1) ‘nature’ of evidence on the topic and time for which use it is most suited (for different purposes and audiences and at different times); how the evidence is (2) ‘perceived’ and whether it is deemed to constitute robust and useful evidence by stakeholders and the level of (3) ‘demand’ for such evidence by stakeholders and the ease with which it can be ‘supplied’.

Alongside evidence, the four other key concepts in the framework (actors, process, context and approach) represent barriers and facilitators arising from the environment in which evidence is to be used. ‘Actors’, ‘process’ and ‘approach’ form a triangle linked to the factors relating to evidence (perception, supply and demand, and use).

‘Context’, while distinct, permeates all other concepts and is therefore displayed as a triangle housing the framework. The outer sides of the triangle denote the three interlinking sublevels of ‘context’: ‘micro’ (individuals), ‘meso’ (organizations) and ‘macro’ (systems). The positioning of actors, process and approach at the corners of the triangle highlights the most pertinent links between these concepts and the sublevels of context. ‘Actors’ sit at the intersection of ‘micro’ and ‘meso context’ because *actors* engage with agenda-setting as individuals and through their organizational role. ‘Approach’ sits at the intersection of ‘micro’ and ‘macro context’ because ‘approach’ involves individuals seeking to have a systemic impact, often through their organizational role. ‘Process’ sits at the intersection of ‘meso’ and ‘macro context’ because ‘process’ involves organizations and therefore individuals within these organization, working within systems.

Arrows, indicating cross-cutting dimensions (beliefs, values and interests; capacity; power and politics; and trust and relationships) link the five concepts (evidence, actors, process, context and approach). Whilst initially falling under the five concepts, as the analysis proceeded it became clear that these dimensions were apparent under several, if not all, of the concepts. Only the most pertinent links are displayed in the framework; all the concepts link together in complex, myriad ways. Furthermore, all of the concepts are linked through each other, for example, capacity influences the approach via actors. The use of double-headed arrows indicates the bi-directional influence, which are now explained.

‘Actors’ can ‘perceive evidence’ differently due to the nature of their personal, professional and/or cultural positioning with respect to that ‘evidence’. On the other hand, the ways in which ‘actors’ relate to ‘evidence’, such as the role they play in the policy process, can also be influenced by their ‘perception of evidence’. The agenda-setting ‘process’ influences the ‘demand’ for and consequent ‘supply of evidence’. On the other hand, the ‘supply’ of evidence can also influence the agenda-setting ‘process’. Appropriate ‘approaches’ to strengthening the use of evidence in agenda-setting are influenced by the intended ‘use of evidence’ in agenda-setting. On the other hand, ‘approach’ can also influence how ‘evidence is used’. Evidence, such as community narratives may, for example, be used to drive interest in mental health policy issues, leading to demands for further evidence.

While *‘*context’ influences all aspects of ‘evidence’ in policy agenda-setting, the predominant influence is via the ‘beliefs, values and interests’ of actors as individuals (‘micro context’) and through their organizational role (‘meso context’). On the other hand, the ‘beliefs, values, and interests’ of ‘actors’ also influence the ‘context’ in which agenda-setting is undertaken. For mental health, the surrounding stigma can influence public opinion and the level of political attention received.

The extent of ‘trust’, and nature of the ‘relationships’ between ‘actors’, influences the extent to which ‘approach’ can be effective in strengthening the ‘use of evidence’. Some approaches may be dependent upon trusted relationships, for example, the use of knowledge intermediaries to share evidence. On the other hand, the kind of ‘approach’ used can influence the extent of ‘trust’ and nature of the’ relationships’ developed between ‘actors’. Developing relationships between actors, for example, by providing informal networking opportunities, may be an integral component of an approach, The policy ‘process’ is inherently ‘political’, and deciding the ‘approach’ needs to take into account the ‘power’ dynamics at play. On the other hand, the ‘approach’ taken can influence the distribution of ‘power’ in the policy ‘process’. ‘Actors’ capacity’ is a key determinant of their involvement in the policy ‘process’. On the other hand, involvement in the policy ‘process’ can magnify ‘actors’ capacity’ to engage, such as through increasing their experience and skills.

## Discussion

In this article, we reported the analysis of (reviews of) frameworks that explain evidence-informed policy-making and proposed a resultant meta-framework for evidence-informed agenda-setting for mental health policies.

Our meta-framework complements the existing body of knowledge and advances the literature through collating in a novel way a vast body of relevant information. One area we advance the knowledge on evidence-informed policy-making is a deeper understanding of the context of, and approach to, evidence-informed mental health policy-making. For example, issues such as trust, power and capacity permeate across the micro, meso and macro levels of the context and can often be intrinsically linked. We also advance the theorization of evidence-informed policy-making through highlighting the distinction of approach and process of evidence use, which need to be examined separately, offering useful practical insights for stakeholders working towards strengthening the use of evidence. Effective approaches may incorporate a broader range of activities and actors than is necessarily apparent from a focus on how policies are made and the flow of evidence. The additional focus on how the concepts are linked together offers an insight into the more diverse, and often indirect, ways in which evidence may be used to inform health policy-making.

Although frameworks by their nature are a simplification, a criticism is that current frameworks treat the use of health evidence in policy as a ‘black box’ (Gold, 2009). A single framework is unlikely to be able to unpack the required complexity for all contexts and use cases; our framework focuses on some previously underexplored areas. Mental health, including as a policy issue, has been argued to be a ‘wicked problem’ that is inherently complex (Hannigan and Coffey, 2011). Mental health differs from other health policy issues; despite the recent calls for greater integration in research, policy and practice (Collins *et al.*, 2013), mental health is still often considered separately to physical health, with the aim of delivering mental health services that are as good as those for physical health rather than as part of health services (Naylor *et al.*, 2016). Evidence for mental health is also polarizing, with a lack of a global consensus on the classification, cause and treatment of mental health (Mackenzie, 2014). In LMICs, these are even more contentions, with further criticisms of top-down impositions of Western models of mental illness (Whitley, 2015).

Our meta-framework aims to consider and incorporate some of this complexity through the four cross-cutting dimensions. Reviews have noted the increasing value in including ‘software’ elements of health systems (e.g. beliefs, values and interests) alongside their ‘hardware’ elements (e.g. human and financial resources). However, the social and political context of decision-making, the next layer in representing the complexity of health policy and systems (Sheikh *et al.*, 2011), has been identified as the next area of development for evidence-to-policy frameworks. The four cross-cutting dimensions therefore incorporate the soft factors into the meta-framework, as well as the social and political context to highlight areas for further research.

### Key issues for mental health agenda-setting in LMICs

According to the findings of this review, we can observe three key issues important in aiding application and in advancing evidence-informed mental health agenda-setting in LMICs.

First, our findings call for greater attention to be given to informal evidence, evidence based on personal experience, e.g. expert opinion and stakeholder consultations (Mbachu *et al.*, 2016). This echoes calls by other authors for evidence-based health policy research to consider a broader definition of evidence (Oliver *et al.*, 2014). This is a particularly poignant finding for this review as the only framework developed for mental health agenda-setting in LMICs exclusively focuses on formal scientific evidence (Votruba *et al.*, 2020; 2021).

Our study complements, and extends, the existing EVITA framework for mental health agenda-setting in LMICs (Votruba *et al.*, 2020; 2021) by expanding the scope of our framework to explicitly include informal evidence. Several reviews identified a need to understand how research is combined with other forms of knowledge, with some recognition of tacit (that is difficult to codify) knowledge being important alongside explicit knowledge. However, formal research evidence tended to be the predominant, sometimes implicit, focus. According to the findings of this review, we can observe that a further distinction of explicit knowledge, between formal research evidence, and informal evidence is likely to be useful to capture context-specific experiences, which are often undocumented and unpublished but can be equially influential for agenda-setting. For mental health LMIC contexts, this is particularly pertinent as formal evidence is often less abundant. Relevant knowledge resides outside formal channels, for example, with individuals and organizations at the grassroots level, thus highlighting the importance of informal evidence. Furthermore, the only framework aimed at mental health agenda-setting in LMICs identified exclusively focuses on formal research evidence.

The limited focus on the role of informal evidence also often extends to policy analysis of existing policies, which often focuses on formal research evidence (Bhugra *et al.*, 2018), presumably due to methodological challenges. Furthermore, because as argued by Greenhalgh and Russell (2009)—research evidence can inform, but not determine, political decision-making, where value-based decisions about ‘what to do’ are needed. Informal evidence based on personal experiences may therefore be a key consideration for agenda-setting in LMICs where there are multiple competing demands. Inclusion of more diverse types of evidence does not just broaden the scope of the framework but influences all the components; the nature of the evidence sits at the centre of the framework. Our framework is suited to a different use case, which is not limited to direct, ‘top-down’ use of evidence, but that also recognizes a more ‘bottom-up’ use of evidence, including by the different policy actors.

Second, our review highlights the importance of communities. Frameworks mostly focus on the ‘two communities’ of researchers and policymakers, and, increasingly, intermediaries who attempt to bridge this gap (Tantivess and Walt, 2008). Policymaker is a broad category and is often used ambiguously (MacKillop *et al.*, 2020); due to the importance of the receivers of evidence highlighted by this review, this term would benefit from distilling.

While we reinforce the importance of capacity and relationships between different policy actors acknowledged elsewhere (e.g. Green and Bennett, 2007; Hawkes *et al.*, 2016; Oronje *et al.*, 2019; Votruba *et al.*, 2020), our findings go further and suggest that a broader range of actors should be considered to maximize fully the use of broader range of evidence to inform policy-making. Our findings also echo the recognized importance of a wide range of stakeholders, for each stage in the policy process, including agenda-setting (World Health Organization, 2005). Other scholars have also argued that it is important to consider all relevant mental health policy stakeholders as they may have the potential of introducing policy windows or barriers (Makan *et al.*, 2015).

Nascent frameworks are beginning to include a broader array of actors, including advocacy coalitions and included enactors, or those actors who are engaged in either research or policy processes (Votruba *et al.*, 2020; 2021). We argue that further broadening the scope of the stakeholders to include those not already engaged is necessary to ensure those marginalized are not further excluded in agenda-setting and that any agenda is co-created.

Involvement of a greater range of actors in promoting the use of evidence in agenda-setting would be expected to lead to a more indirect flow of evidence from researchers to policymakers, broadening the range of potential approaches. Recent attention to the importance of communities for strengthening the use of evidence for global health policies has been evoked by the COVID-19 pandemic (AlKhaldi *et al.*, 2021).

Bidirectionality should be a key component of their inclusion in frameworks, given the importance of genuine engagement (Conklin *et al.*, 2010). However, the real-world practicalities of such an endeavour are challenging (Tebaldi *et al.*, 2017). Due to the likely differences across actors, the recommendation by Oliver et al. (2014) to understand the daily lives of individuals to understand how they use evidence is likely to be of greater significance.

Widening the range of actors considered in frameworks is particularly important for LMIC settings. As argued by Malekinejad et al. (2018), the role of intermediaries and advocates is especially important for marginalized communities, such as the working poor and undocumented migrants, who are often neglected in the policy agenda, and hence service delivery. The importance of advocates is compounded for mental health by the stigmatization that surrounds the topic and of those affected (Malekinejad *et al.*, 2018). Additionally, in LMICs, a significant proportion of health treatment occurs in the informal sector, including for mental health (Mackenzie, 2014), again broadening the range of stakeholders. Furthermore, decentralization has featured in the health sector reforms of a majority of LMICs (Cobos Muñoz *et al.*, 2017), which has been argued to lead to exponential growth in participation of citizens in decision-making processes, including in Brazil (Suárez, 2006).

Different actors, however, often do not have the same power. People with lived experience of mental conditions, recognized as important participants, may face barriers to engaging in policy processes due to their health status (Abayneh *et al.*, 2017). A lack of treatment and support can reduce the motivation and ability of service users to engage (Kleintjes *et al.*, 2010). However, some authors simultaneously caution that the role of communities should equally not be overstated to unduly burden resource constrained groups and people (Tebaldi *et al.*, 2017).

Third, although our results do not directly highlight this, our reflections on the results of our review highlight the importance of distinguishing policy agenda-setting from routine practices such as service delivery. Policy is often grouped with practice by reviews (e.g. Milat and Li, 2017) and frameworks. Although inter-related, and changes in practice are the ultimate aim of policy change, policy and practice are distinct (Jansen *et al.*, 2010). A criticism levelled at the health evidence-to-policy literature is that policy theory, and knowledge of the policy process, is seldom used (Cairney and Oliver, 2017). Frameworks that consider policy and practice could be expected to utilize theory and knowledge related to policy less. Cairney and Oliver (2017) highlight that evidence is valued and used differently in evidence-based policy and evidence-based medicine. In addition, not solely focusing on policy may lead to less consideration of the different stages of the policy cycle, missing much of the complexity (Oliver *et al.*, 2014). Moreover, a focus on policy will facilitate a greater focus on the political nature of policy-making, and the role of power that is especially pertinent for mental health, which is often shied away from. Our meta-framework, and the accompanying questions we have developed as a guide outlined next, focuses on policy, and specifically on agenda-setting, allowing us to delve deeper into any idiosyncrasies.

### Key considerations for application of the framework

Accordingly, from the key issues identified for mental health agenda-setting in LMICs in relation to the meta-framework, a list of five accompanying questions was developed (Table 5). The questions, and suggested considerations, are intended as a guide for thinking about the components identified in the meta-framework (evidence, actors, process, context and approach) with relevance to mental health agenda-setting in LMICs. They enable more specific application of the framework for the setting to which the framework might be applied.

**Table 5.** Questions to accompany the meta-framework

	Key question	Considerations
Evidence	What is the nature of the evidence that can be used to inform agenda-setting?	Formal research evidence and/or informal evidence based on personal experience.
Actors	To which stakeholder group(s) is the evidence directed?	Stakeholders already engaged in mental health agenda-setting and/or those new to this? Co-creating mental health agenda-setting, including with communities and marginalized groups.
Process	In what ways can different stakeholder groups use the evidence to influence agenda-setting?	Directly and/or indirectly.
Context	How will contextual factors affect the use of evidence by the stakeholders identified?	At the level of the individual, organization and system.
Approach	How can the approach be tailored for the evidence and stakeholders identified?	Consider issues of trust and relationships; capacity; power and politics; and beliefs, values and interests.

### Study limitations

Due to the large number of existing frameworks, and the diverse terminology used, it is possible that some relevant reviews, and frameworks, were missed. However, although the reviews differed slightly in their focus, they had broadly similar findings. Due to the large number of frameworks included within the reviews, it was not possible to analyse all the frameworks individually, and the analysis of the authors of the reviews had to be relied upon. To mitigate this, individual sources were followed up, where needed. The large proportion of shared findings between the reviews also suggested robustness of the analysis of the reviews.

Only English language reviews were included, and consequently, some relevant reviews may have been excluded, a limitation exacerbated by most of the included reviews themselves only including frameworks from English language publications. As highlighted by Almeida and Báscolo (2006), translation can critically alter meaning. Given the low proportion of health research published on LMICs originating from local authors (Busse and August, 2020), a trend that has also been observed for mental health (Razzouk *et al.*, 2010), key factors influencing the role of evidence in mental health agenda-setting in LMICs may be overlooked by current frameworks. Furthermore, the position of the authors as researchers and professional actors, albeit with a range of experiences, may have influenced the analysis of the results and the development of the framework. Future work collaborating with stakeholders often marginalized from policy-making and agenda-setting processes, including communities, to refine the framework and thus inform that approaches to strengthening the use of evidence for mental health agenda-setting would be beneficial.

## Conclusions

Our review has built upon the multitude of evidence-to-policy frameworks by collating the literature in a novel way. Consequently, our resultant meta-framework enables a deeper understanding of the context of and approach to evidence-informed mental health agenda-setting, for which there has been limited attention to date. Only one framework was found that focuses on this aspect, which we build upon and extend. Furthermore, we advance theory by distinguishing between approach and process of evidence use, which is of use for stakeholders working to strengthen the use of evidence. The current health frameworks were critically analysed from the perspective of mental health agenda-setting in LMICs to develop recommendations of how current frameworks could be further developed to be tailored to this specific context. Our expanded focus on what constitutes evidence and whom it is used by for mental health agenda-setting in LMICs, in addition to the conventional contribution of formal research, offers unique insights for strengthening the use of evidence. Our expanded focus aims to consider facilitating and accruing the contribution of the context and voices of individuals and stakeholders towards shaping and impacting the policy agenda.

## Supplementary Material

czad038_Supp

## Data Availability

The data underlying this article are available in the article and in its online supplementary material.

## Reflexivity statement

The authors represent a range of locations, seniority and gender identities. Authors are based in India and the UK, representing both lower-middle-income countries and high-income countries. Most authors are senior academics, but the first author was a PhD student when the research was taking place. A majority, three out of the five authors identify as women, with the remaining authors identifying as men.
