# Supplementary material for: Using evidence in mental health policy agenda-setting in low- and middle-income countries: a conceptual meta-framework from a scoping umbrella review
Source: Health Policy Plan. 2023 Jun 17;38(7):876–93. doi: 10.1093/heapol/czad038 (PMC10394497; doi:10.1093/heapol/czad038)
Supplement: czad038_Supp [file czad038_supp.zip › suppl_data/SI 1.docx]

Supplementary Information 1. Search strategy and results

Ovid MEDLINE(R) 1996 to October Week 5 2018

Date of search 12/11/2018

| Cluster |  | Search Term | Number of Results |
| --- | --- | --- | --- |
| 1 - Theories and Frameworks |  |  |  |
|  | 1 | theor*.tw. | 301,333 |
|  | 2 | model.tw. | 1,307,619 |
|  | 3 | framework.tw. | 140,601 |
|  | 4 | concept.tw. | 142,821 |
|  | 5 | tool.tw. | 312,365 |
|  | 6 | 1 or 2 or 3 or 4 or 5 | 1,987,636 |
|  |  |  |  |
| 2 - Evidence |  |  |  |
|  | 7 | evidence.tw | 1,086,850 |
|  | 8 | research.tw | 888,640 |
|  | 9 | knowledge.tw | 433,381 |
|  | 10 | 7 or 8 or 9 | 2,172,402 |
|  |  |  |  |
| 3 - Policy | 11 | policy mak*.tw. | 16,940 |
|  | 12 | decision mak*.tw. | 88,439 |
|  | 13 | POLICY MAKING/ | 12,616 |
|  | 14 | 11 or 12 or 13 | 113,914 |
|  | 15 | health.tw | 1,125,232 |
|  | 16 | 14 and 15 | 39,842 |
|  |  |  |  |
| 4 - Combine Clusters |  |  |  |
|  | 17 | 6 and 10 and 16 | 6,600 |
|  |  |  |  |
| 5 - Key Phrases |  |  |  |
|  | 18 | (evidence adj based adj policy).tw. | 521 |
|  | 19 | (evidence adj informed adj policy).tw. | 107 |
|  | 20 | (evidence adj to adj decision adj making).tw. | 1,053 |
|  | 21 | (research adj3 policy adj3 pathway*).tw. | 5 |
|  | 22 | (getting adj3 research adj3 into adj3 policy adj3 practice).tw. | 5 |
|  |  |  |  |
| 6 – Combine all searches |  |  |  |
|  | 23 | 17 or 18 or 19 or 20 or 21 or 22 | 7,974 |
|  |  |  |  |
| 7 - Additional Limits |  |  |  |
| Limit to humans and English language | 24 | Limit 23 to (humans and english language) | 7,127 |
| Limit to 2004 – Current | 25 | limit 24 to yr="2004 -Current" | 6,356 |
| Limit to Review Articles | 26 | limit 25 to "review articles" | 1,456 |

Global Health 1973 to 2018 Week 44

Date of search 12/11/2018

| Cluster |  | Search Term | Number of Results |
| --- | --- | --- | --- |
| 1 - Theories and Frameworks |  |  |  |
|  | 1 | theor*.tw. | 39,843 |
|  | 2 | model.tw. | 223,817 |
|  | 3 | framework.tw. | 27,604 |
|  | 4 | concept.tw. | 23,416 |
|  | 5 | tool.tw. | 52,778 |
|  | 6 | 1 or 2 or 3 or 4 or 5 | 336,137 |
|  |  |  |  |
| 2 - Evidence |  |  |  |
|  | 7 | evidence.tw | 202,110 |
|  | 8 | research.tw | 257,631 |
|  | 9 | knowledge.tw | 112,194 |
|  | 10 | 7 or 8 or 9 | 510,174 |
|  |  |  |  |
| 3 - Policy | 11 | policy mak*.tw. | 9477 |
|  | 12 | decision mak*.tw. | 17,386 |
|  | 13 | HEALTH POLICY/ | 17,539 |
|  | 14 | 11 or 12 or 13 | 40,987 |
|  | 15 | health.tw | 621,781 |
|  | 16 | 14 and 15 | 32,429 |
|  |  |  |  |
| 4 - Combine Clusters |  |  |  |
|  | 17 | 6 and 10 and 16 | 4,222 |
|  |  |  |  |
| 5 - Key Phrases |  |  |  |
|  | 18 | (evidence adj based adj policy).tw. | 263 |
|  | 19 | (evidence adj informed adj policy).tw. | 78 |
|  | 20 | (evidence adj to adj decision adj making).tw. | 349 |
|  | 21 | (research adj3 policy adj3 pathway*).tw. | 4 |
|  | 22 | (getting adj3 research adj3 into adj3 policy adj3 practice).tw. | 9 |
|  |  |  |  |
| 6 – Combine all searches |  |  |  |
|  | 23 | 17 or 18 or 19 or 20 or 21 or 22 | 4,719 |
|  |  |  |  |
| 7 - Additional Limits |  |  |  |
| Limit to English language (limit to humans not available) | 24 | limit 23 to english language | 4,425 |
| Limit to 2004 – Current | 25 | limit 24 to yr="2004 -Current" | 4,243 |
| Review Articles | 26 | review.mp. or literature reviews/ or systematic reviews/ or reviews/ | 264,446 |
|  |  | 25 and 26 | 1054 |

PsycINFO 2002 to November Week 1 2018

Date of search 12/11/2018

| Cluster |  | Search Term | Number of Results |
| --- | --- | --- | --- |
| 1 - Theories and Frameworks |  |  |  |
|  | 1 | theor*.tw. | 387,690 |
|  | 2 | model.tw. | 340,157 |
|  | 3 | framework.tw. | 125,643 |
|  | 4 | concept.tw. | 86,863 |
|  | 5 | tool.tw. | 68,558 |
|  | 6 | 1 or 2 or 3 or 4 or 5 | 796906 |
|  |  |  |  |
| 2 - Evidence |  |  |  |
|  | 7 | evidence.tw | 325,021 |
|  | 8 | research.tw | 716,922, |
|  | 9 | knowledge.tw | 202,603 |
|  | 10 | 7 or 8 or 9 | 1,042,458 |
|  |  |  |  |
| 3 - Policy | 11 | policy mak*.tw. | 18,636 |
|  | 12 | decision mak*.tw. | 72,303 |
|  | 13 | POLICY MAKING/ | 15,415 |
|  | 14 | 11 or 12 or 13 | 99,528 |
|  | 15 | health.tw | 421,489 |
|  | 16 | 14 and 15 | 22,412 |
|  |  |  |  |
| 4 - Combine Clusters |  |  |  |
|  | 17 | 6 and 10 and 16 | 4,962 |
|  |  |  |  |
| 5 - Key Phrases |  |  |  |
|  | 18 | (evidence adj based adj policy).tw. | 441 |
|  | 19 | (evidence adj informed adj policy).tw. | 60 |
|  | 20 | (evidence adj to adj decision adj making).tw. | 341 |
|  | 21 | (research adj3 policy adj3 pathway*).tw. | 2 |
|  | 22 | (getting adj3 research adj3 into adj3 policy adj3 practice).tw. | 2 |
|  |  |  |  |
| 6 – Combine all searches |  |  |  |
|  | 23 | 17 or 18 or 19 or 20 or 21 or 22 | 5,706 |
|  |  |  |  |
| 7 - Additional Limits |  |  |  |
| Limit to humans and English language | 24 | Limit 23 to (human and english language) | 5,474 |
| Limit to 2004 – Current | 25 | limit 24 to yr="2004 -Current" | 5,330 |
| Limit to Review Articles | 26 | exp "LITERATURE REVIEW"/ or review.mp. | 1,456 |
|  | 27 | 25 and 26 | 932 |
| Limit to Peer-review | 28 | limit 27 to peer reviewed journal | 632 |

HMIC Health Management Information Consortium 1983 – present

Date of search 12/11/2018

| Cluster |  | Search Term | Number of Results |
| --- | --- | --- | --- |
| 1 - Theories and Frameworks |  |  |  |
|  | 1 | theor*.tw. | 8,932 |
|  | 2 | model.tw. | 15,440 |
|  | 3 | framework.tw. | 12,735 |
|  | 4 | concept.tw. | 5,216 |
|  | 5 | tool.tw. | 4,786 |
|  | 6 | 1 or 2 or 3 or 4 or 5 | 40,656 |
|  |  |  |  |
| 2 - Evidence |  |  |  |
|  | 7 | evidence.tw | 28,294 |
|  | 8 | research.tw | 39,509 |
|  | 9 | knowledge.tw | 11,574 |
|  | 10 | 7 or 8 or 9 | 67,205 |
|  |  |  |  |
| 3 - Policy | 11 | policy mak*.tw. | 3342 |
|  | 12 | decision mak*.tw. | 6957 |
|  | 13 | POLICY MAKING/ | 1575 |
|  | 14 | 11 or 12 or 13 | 11,075 |
|  | 15 | health.tw | 139,791 |
|  | 16 | 14 and 15 | 6,657 |
|  |  |  |  |
| 4 - Combine Clusters |  |  |  |
|  | 17 | 6 and 10 and 16 | 1,047 |
|  |  |  |  |
| 5 - Key Phrases |  |  |  |
|  | 18 | (evidence adj based adj policy).tw. | 185 |
|  | 19 | (evidence adj informed adj policy).tw. | 21 |
|  | 20 | (evidence adj to adj decision adj making).tw. | 128 |
|  | 21 | (research adj3 policy adj3 pathway*).tw. | 0 |
|  | 22 | (getting adj3 research adj3 into adj3 policy adj3 practice).tw. | 0 |
|  |  |  |  |
| 6 – Combine all searches |  |  |  |
|  | 23 | 17 or 18 or 19 or 20 or 21 or 22 | 1,316 |
|  |  |  |  |
| 7 - Additional Limits |  |  |  |
| Limit to English language (limit to humans not available) | 24 | Limit 23 to english language | 1,243 |
| Limit to 2004 – Current | 25 | limit 24 to yr="2004 -Current" | 838 |
| Limit to Review Articles | 26 | review.mp. | 28,138 |
|  | 27 | 25 and 26 | 189 |
