# Supplementary material for: Using evidence in mental health policy agenda-setting in low- and middle-income countries: a conceptual meta-framework from a scoping umbrella review
Source: Health Policy Plan. 2023 Jun 17;38(7):876–93. doi: 10.1093/heapol/czad038 (PMC10394497; doi:10.1093/heapol/czad038)
Supplement: czad038_Supp [file czad038_supp.zip › suppl_data/SI 2.docx]

Supplementary Information 2. Adapted GRADE CERQual tool

Overall CERQual Evidence Profile

| **Summary of review finding** |  |
| --- | --- |
| **Studies contributing to the review finding** |  |
| **Methodological limitations** | No or very minor/  Minor/  Moderate/  Serious concerns |
| **Coherence** | No or very minor/  Minor/  Moderate/  Serious concerns |
| **Adequacy** | No or very minor/  Minor/  Moderate/  Serious concerns |
| **Relevance** | No or very minor/  Minor/  Moderate/  Serious concerns |
| ***CERQual assessment of confidence in the evidence*** |  |
| **Explanation of CERQual assessment** |  |

Assessment of Methodological Limitations

| **Question** | **Assessment** | **Comments** |
| --- | --- | --- |
| **1. Did the review address a clearly defined question?** | Yes  No  Can’t answer  Not applicable |  |
| **2. Was there duplicate study selection and data extraction?**  There should be at least two independent data extractors and a consensus procedure for disagreements should be in place. | Yes  No  Can’t answer  Not applicable |  |
| **3. Was a comprehensive literature search performed?**  At least two electronic sources should be searched. The report should include years and databases used. Key words and/or MESH terms should be stated and where feasible the search strategy should be provided. All searches should be supplemented by consulting current contents, reviews, textbooks, specialized registers, or experts in the field of study, and by reviewing the references in the studies found. | Yes  No  Can’t answer  Not applicable |  |
| **5. Were the inclusion and exclusion criteria explicitly stated?** | Yes  No  Can’t answer  Not applicable |  |
| **6. Was a list of studies (included and excluded) provided, with justifications given?** | Yes  No  Can’t answer  Not applicable |  |
| **7. Do you think all the important, relevant studies were identified and/or included?**  The search strategy should be designed to identify studies that would best address the review’s question. Are there any key frameworks that have not been included? | Yes  No  Can’t answer  Not applicable |  |
| **8. Was the risk of selection bias assessed?** | Yes  No  Can’t answer  Not applicable |  |
| **9. Were the characteristics of the included studies provided, with adequate detail?**  In an aggregated form such as a table, data from the original studies should be provided. | Yes  No  Can’t answer  Not applicable |  |
| **10. Did the review’s authors do enough to assess and document quality of the included studies?** | Yes  No  Can’t answer  Not applicable |  |
| **11. Did the review authors report on the sources of funding for the studies included in the review?** | Yes  No  Can’t answer  Not applicable |  |
| **12. Were the methods used to synthesise the findings of studies appropriate?** | Yes  No  Can’t answer  Not applicable |  |
| **13. Was the quality of the included studies used appropriately in formulating conclusions?**  The results of the methodological rigor and quality should be considered in the analysis and the conclusions of the review, and explicitly stated in formulating recommendations. | Yes  No  Can’t answer  Not applicable |  |
| **14. Are the overall results of the review clearly stated?** | Yes  No  Can’t answer  Not applicable |  |
| **15. Was the conflict of interest stated?**  Potential sources of support should be clearly acknowledged | Yes  No  Can’t answer  Not applicable |  |
|  |  |  |
| **Overall assessment of methodological limitations** | No or very minor concerns  Minor concerns  Moderate concerns  Serious concerns |  |

Coherence

| Question | Assessment | Comments |
| --- | --- | --- |
| **1. Is the fit between the underlying data from the primary studies and the review finding clear and cogent?**  The nature of the review finding affects the threats to coherence that a review finding faces. The coherence of a descriptive finding may be threatened, if it only describes the most dominant patterns in the data and does not sufficiently capture the presence of ‘outliers’ and/or ambiguous elements in the data. The coherence of an interpretive or explanatory finding may be threatened by the presence of data in the primary studies that challenge the main interpretation or explanation in the review finding (‘disconfirming cases’) or by plausible competing interpretations or explanations. | Contradictory data (explanatory review finding)  Ambiguous or incomplete data (descriptive review findings)  Plausible alternative descriptions, interpretations or explanations (descriptive or explanatory review findings) |  |
| **2. If theory is used to explain underlying patterns in the data, what is the source of the theory?** | Imported (external to papers included in the synthesis)  Developed from the theory used in paper(s) included in the synthesis and then applied across findings from other papers  Theories developed as an original explanation or interpretation |  |
| **3. What is the utility of the review?**  There is a trade-off between coherence and utility. Review authors could, for example, strengthen the coherence of a review finding by reframing it in more general, vague or equivocal terms, or alternatively, in a highly specified fashion such that it only applies to a very limited number of cases. |  |  |
|  |  |  |
| **Overall assessment of coherence** | No or very minor concerns  Minor concerns  Moderate concerns  Serious concerns |  |

Adequacy of Data

| Question | Assessment | Comments |
| --- | --- | --- |
| **1. Demands on data quantity**  The scope of a review finding affects the demands on data quantity | Review finding with limited scope - fewer demands on data quantity  Review finding with broad scope - more demands on data quantity |  |
| **2. Data quantity**  Number of frameworks |  |  |
| **3. Demands on data richness**  The nature of a review finding affects the demands on data richness | Descriptive review finding - fewer demands on data richness  Explanatory review finding - more demands on data richness |  |
| **4**. **Data richness**  Does the data provide you with sufficient details to gain an understanding of the phenomenon described in the review finding. | Yes  No  Can’t answer  Not applicable |  |
|  |  |  |
| **Overall assessment of data adequacy** | No or very minor concerns  Minor concerns  Moderate concerns  Serious concerns |  |

Relevancy of Data

| Question | Assessment | Comments |
| --- | --- | --- |
| **1. Dimensions of context to consider as specified in the question and protocol** |  |  |
| **2. Assessment of relevance of the studies contributing to the finding mapped against the review question context** | Direct Relevance  Indirect Relevance  Partial Relevance  Uncertain Relevance |  |
|  |  |  |
| **Overall assessment of Data Relevancy** | No or very minor concerns  Minor concerns  Moderate concerns  Serious concerns |  |
