# Supplementary material for: Using evidence in mental health policy agenda-setting in low- and middle-income countries: a conceptual meta-framework from a scoping umbrella review
Source: Health Policy Plan. 2023 Jun 17;38(7):876–93. doi: 10.1093/heapol/czad038 (PMC10394497; doi:10.1093/heapol/czad038)
Supplement: czad038_Supp [file czad038_supp.zip › suppl_data/SI 3.docx]

Supplementary Information 3. Summary of included reviews

| ***N*** | **Author (year)** | **Nature of the review** | **Scope of the review** | **Name of framework (if produced)** | **Key elements of the framework (or synthesis)** | **Contribution to knowledge** | **Level of confidence (GRADE-CerQual** |
| --- | --- | --- | --- | --- | --- | --- | --- |
| ***1*** | Contandriopoulos et al., 2010 | (Narrative) Systematic review | Knowledge exchange processes at the organizational and policymaking levels | An integrative model of collective-level knowledge transfer | Three components of knowledge exchange systems: the roles of individual actors working in collective systems, the nature of the knowledge exchanged, and the process of knowledge use. Based on three dimensions of context: politics, economics, and social structuring. | Aids in understanding the components (actors, evidence and process) of research-to-policy pathways and what contextual factors are important at the policy-making level. | Low |
| ***2*** | Damschroder et al., 2009 | Non-systematic review | Models, theories, and frameworks that facilitate translation of research findings into practice primarily within the healthcare sector | Consolidated Framework for Implementation Research (CFIR) | Five major domains: intervention characteristics, outer setting, inner setting, characteristics of the individuals involved, and the process of implementation. | Aids in understanding what influences the implementation stage of the policy cycle. | Moderate |
| ***3*** | Gold, 2009 | Non-systematic review | Policy formation and organizational behavior, factors influencing research use, and knowledge transfer and exchange strategies | Factors, processes, and actors that shape pathways between research and its use | Three types of pathways to research translation: (1) research findings drive use; (2) effective intermediaries convert research to policy information; and (3) user seeks to influence or enhance value of research | Helps to understand, more explicitly, on the potential pathways that link research to policy makers' decision-making applications, and the barriers to these pathways. | Low |
| ***4*** | Graham et al., 2006; Graham et al., 2007 | Non-systematic review | Knowledge translation planned action theories, models and frameworks for research to policy and practice. | Action categories representing steps of a planned action model; The Knowledge-to-action process framework | 1) nature  of the evidence or knowledge (n = 24), 2) attributes of  change or innovation (n = 21), 3) who the audience is  (n = 28), 4) organizational context and culture in which  the change is to take place (n = 28), 5) the organizational  resources and support for the proposed change (n = 25),  and 6) implementation-related factors (n = 28).  1) nature  of the evidence or knowledge (n = 24), 2) attributes of  change or innovation (n = 21), 3) who the audience is  (n = 28), 4) organizational context and culture in which  the change is to take place (n = 28), 5) the organizational  resources and support for the proposed change (n = 25),  and 6) implementation-related factors (n = 28).  Knowledge-to-action process involves knowledge creation, and a cycle of activities and processes related to use or application of knowledge. Key factors: nature of the evidence or knowledge; attributes of change or innovation; who the audience is; organizational context and culture in which the change is to take place; the organizational resources and support for the proposed change; implementation-related factors. | Aids in understanding the different stages of research-to-policy pathways | Very low |
| ***5*** | Green et al., 2009(L. W. Green et al., 2009) | Non-systematic review | Diffusion dissemination, and implementation aspects of research translation in public health practice and community change | Utilisation-focused surveillance framework | (1) The source of data should be credible and involve potential users early in the research process; (2) the content of data should be accessible, adaptable, and sound; (3) the medium of transmission should be tailored and multiple in sources; (4) the user should be supported in knowledge acquisition and the implications of change implied by the data; and (5) the context of use can provide incentives and leadership in utilisation. | Aids in understanding what factors influence the general research-to-action pathway, without an explicit focus on policy. | Low |
| ***6*** | Greenhalgh, et al., 2007; Greenhalgh et al., 2004 | Systematic review | How innovations can be spread and sustained in health service delivery and organizations. | Conceptual Model for Considering the Determinants of Diffusion, Dissemination, and Implementation of Innovations in Health Service Delivery and Organization | The model considers both content (defining and measuring the diffusion of innovation in organizations) and process: the innovation; adoption by individuals; assimilation by the system; diffusion and dissemination; system antecendents for innovation; system readiness for innovation; the outer context (interorganizational networks and collaboration); implementation and routinization. Linkage among components of the model is also considered. | Aids in understanding  the implementation stage of the policy cycle, and how new evidence-based policy solutions can spread. | High |
| ***7*** | Moullin et al., 2015 | Systematic review | Implementation frameworks of innovations in healthcare | A Generic implementation framework (GIF) | The framework includes the process of implementation (often portrayed as a series of stages and/or steps), the innovation to be implemented, the context in which the implementation is to occur (divided into a range of domains), and influencing factors, strategies, and evaluations. | Aids in understanding the implementation stage of the policy cycle. | High |
| ***8*** | Votruba et al., 2018 | Systematic Review | Theories, frameworks and models to understand and guide action in research evidence and policy interrelationships in mental health and LMIC (a focus on policy agenda-setting was removed due to no results). | A framework for the interrelationship of mental health evidence and policy in low- and middle-income countries | Seven themes identified: political context, external influences, actors, evidence, intermediaries and links, capacity, and catalysts. | Aids in understanding what factors influence the research-to-policy pathway, with a specific focus on mental health in LMIC. | High |
| ***9*** | Ward et al., 2009 | Non-systematic review | Models that explain all or part of the knowledge transfer process | A framework for transferring knowledge into action | Five common components of the knowledge transfer process that are connected via a complex, multidirectional set of interactions: problem identification and communication; knowledge/research development and selection; analysis of context; knowledge transfer activities or interventions; and knowledge/research utilisation. | Aids in understanding what factors influence the general research-to-action pathway, without an explicit focus on policy. | High |
| ***10.*** | Almeida & Báscolo, 2006 | (Critical) Non-systematic review | The theoretical literature on the relationship between research results and its use in policy decision-making, formulation and implementation. | Did not produce a framework | 1) Presents analytical models designed to explain the relationships between the production of scientific knowledge and its use in policy formulation and implementation. 2) Reviews the issue of the use of research results and policymaking. 3) Analyses the literature on the interaction between researchers and policymakers. 4) Reviews issues related to the spread of knowledge, knowledge transfer, and evidence base in health policy and practice. | Aids in understanding the research-to-policy frameworks available | Low |
| ***11.*** | Milat & Li, 2017 | (Narrative) Non-systematic review | Frameworks for translating research evidence into policy and practice | Did not produce a framework | The review 1) examined different research translation frameworks; 2) examined the similarities and differences between the frameworks; and 3) identified key strengths and weaknesses of the models when they are applied in practice. | Aids in understanding the components of research-to-policy and –practice frameworks available, and where they have been applied. | High |
| ***12.*** | Mitchell et al., 2010 | Non-systematic Review | Theoretical models for translational science in nursing | Did not produce a framework | Four thematic areas emerged: (1) evidence-based practice and knowledge transformation processes, (2) strategic change to promote adoption of new knowledge, (3) knowledge exchange and synthesis for application and inquiry, and (4) designing and interpreting dissemination research. | Aids in understanding the components of research-to-policy and –practice frameworks available, and which part of the research-to-policy pathway they focus on. | High |
| ***13.*** | Mitton et al., 2007 | Non-systematic review | The Knowledge, transfer, and exchange (KTE) literature on health care policy. | Did not produce a framework | Emphasizes the importance of knowledge type; the recipient and “receptor capacity”; the messenger; interactions between research producers and the intended user groups and context; the knowledge transfer process and support system; the evaluation strategy, informing debate is a more realistic objective than to change decision‐making outcomes. | Aids in understanding the underlying theory and main components of research-to-policy frameworks, and the barriers and facilitators of these pathways. | High |
| ***14.*** | Nilsen, 2015 | (Narrative) Non-systematic review | Theories, models and frameworks applied in implementation science, including those describing and/or guiding the process of translating research into practice | Did not produce a framework | Proposes five categories of theoretical approaches to achieve three overarching aims: describing and/or guiding the process of translating research into practice (process models); understanding and/or explaining what influences implementation outcomes (determinant frameworks, classic theories, implementation theories); and evaluating implementation (evaluation frameworks). | Aids in understanding the research-to-policy and –practice frameworks available, with a focus on the implementation and evaluation stage. | Low |
| ***15.*** | Oborn et al., 2013 | Non-systematic review | The conceptual landscape around knowledge translation (KT) and how management literature on knowledge and learning theories might inform health services research on KT. | Did not produce a framework | KT has been conceptualised along three competing frames, focusing on: linear transfer of knowledge; KT as a social process; and one that seeks to more fully incorporate contextual issues in understanding research implementation. Three overlapping themes are found in the management literature that inform these debates in the health literature: knowledge boundaries, organisational learning and absorptive capacity. | Aids in understanding how research-to-practice pathways have been conceptualised, and what concepts from the management literature could be included in future frameworks. | Moderate |
| ***16.*** | Tabak et al., 2012 | (Narrative) Non-systematic review | Models for disseminations and implementation research for health | Did not produce a framework | Organised models into 3 categories.  1) Construct flexibility (A five-point scale from broad to operational);  2) Focus on dissemination and/or implementation activities (D/I);  3) The socio-ecological framework. | Aids in understanding the different models available, what parts of the research-to-policy pathways they cover, and where they have been applied. | Moderate |
| ***17.*** | Wilson et al., 2010 | Systematic (scoping) review | Conceptual/organising frameworks relating to research dissemination. | Did not produce a framework | Three predominant theoretical approaches underpinned the frameworks: persuasive communication, diffusion of innovations theory, and social marketing. | Aids in understanding the research-to-policy and –practice frameworks available, and what underlying theories these have been based on. | High |
| ***18.*** | Cruz Rivera et al., 2017 | Systematic review | Methodological frameworks used to measure healthcare research impact, including influence on policymaking | Simplified consolidated methodological framework | Methodological frameworks addressed the influence on policymaking with three key impact categories: the type and nature of policy impact; level of policymaking; and ‘policy networks. | Aids researchers in assessing the impact of research on policy making over different impact categories and timeframes. | High |
| ***19.*** | Newson et al., 2018 | Non-systematic review | Methods for assessing research impacts on policy and the policy utilisation of health research | Descriptive framework for research impact and research use assessments | Compare two key approaches: tracing forward from research and tracing backwards from a policy outcome. Defines the key elements of empirical studies (assessment reason, assessment direction, assessment starting point, unit of analysis, assessment methods, assessment endpoint and outcomes assessed). | Aids researchers in assessing the impact of research on policymaking, and how to  communicate this. | Moderate |
